# Exosomes Released by Influenza-Virus-Infected Cells Carry Factors Capable of Suppressing Immune Defense Genes in Naïve Cells

**DOI:** 10.3390/v14122690

**Published:** 2022-11-30

**Authors:** Yana Zabrodskaya, Marina Plotnikova, Nina Gavrilova, Alexey Lozhkov, Sergey Klotchenko, Artem Kiselev, Vladimir Burdakov, Edward Ramsay, Lada Purvinsh, Marja Egorova, Vera Vysochinskaya, Irina Baranovskaya, Alexandra Brodskaya, Roman Povalikhin, Andrey Vasin

**Affiliations:** 1 Institute of Biomedical Systems and Biotechnology, Peter the Great Saint Petersburg Polytechnic University, 29 Ulitsa Polytechnicheskaya, 194064 St. Petersburg, Russia; 2 Smorodintsev Research Institute of Influenza, 15/17 Ulitsa Professora Popova, 197376 St. Petersburg, Russia; 3 Institute for Quantitative Health Science and Engineering (IQ), Michigan State University, 775 Woodlot Dr, East Lansing, MI 48824, USA; 4 Petersburg Nuclear Physics Institute Named by B. P. Konstantinov of National Research Center, Kurchatov Institute, 1 mkr. Orlova roshcha, 188300 Gatchina, Russia; 5 Saint Petersburg Pasteur Institute, 14 Ulitsa Mira, 197101 St. Petersburg, Russia; 6 Biology Science Department, The University of Chicago, 947 E. 58th St., Chicago, IL 60637, USA; 7 Department of Physiology, Augusta University, 1462 Laney Walker Blvd, CA-3149, Augusta, GA 30912, USA

**Keywords:** exosomes, influenza virus, A549 cells, immunosuppression, exosome isolation

## Abstract

**Background:** Exosomes are involved in intercellular communication and can transfer regulatory molecules between cells. Consequently, they can participate in host immune response regulation. For the influenza A virus (IAV), there is very limited information on changes in exosome composition during cell infection shedding light on the potential role of these extracellular membrane vesicles. Thus, the aim of our work was to study changes in exosomal composition following IAV infection of cells, as well as to evaluate their effect on uninfected cells. **Methods:** To characterize changes in the composition of cellular miRNAs and mRNAs of exosomes during IAV infection of A549 cells, NGS was used, as well as PCR to identify viral genes. Naïve A549 cells were stimulated with infected-cell-secreted exosomes for studying their activity. Changes in the expression of genes associated with the cell’s immune response were shown using PCR. The effect of exosomes on IAV replication was shown in MDCK cells using In-Cell ELISA and PCR of the supernatants. **Results:** A change in the miRNA composition (miR-21-3p, miR-26a-5p, miR-23a-5p, miR-548c-5p) and mRNA composition (*RPL13A*, *MKNK2*, *TRIB3*) of exosomes under the influence of the IAV was shown. Many RNAs were involved in the regulation of the immune response of the cell, mainly by suppressing it. After exosome stimulation of naïve cells, a significant decrease in the expression of genes involved in the immune response was shown (*RIG1*, *IFIT1*, *MDA5*, *COX2*, *NFκB*, *AnxA1*, *PKR*, *IL6*, *IL18*). When infecting MDCK cells, a significant decrease in nucleoprotein levels was observed in the presence of exosomes secreted by mock-infected cells. Viral levels in supernatants also decreased. **Conclusions:** Exosomes secreted by IAV-infected cells could reduce the immune response of neighboring intact cells, leading to more effective IAV replication. This may be associated both with regulatory functions of cellular miRNAs and mRNAs carried by exosomes, or with the presence of viral mRNAs encoding proteins with an immunosuppressive function.

## 1. Introduction

Exosomes are currently being actively studied and applied in the fields of molecular biology, cytology, and biotechnology. Exosomes are nanosized (30–150 nm) membrane vesicles secreted by almost all cell types. As such, they are present in almost all biological fluids, such as urine [1], saliva [2], blood [3], breast milk [4], amniotic fluid [5], and bronchoalveolar fluid [6]. It is known that these nanosized particles contain various types of biomolecules, including proteins, lipids, and carbohydrates, as well as DNA and RNA [7]. An important feature of exosomes is the ability to transfer functional proteins and nucleic acids between cells. Thus, participation in intercellular communication could be considered a key role of exosomes. Exosome composition includes the set and concentrations of proteins, RNA, and other biomolecules. Such composition may vary depending on physiological or pathological intracellular processes [8]. Thus, analysis of exosome composition can be further used in the development of diagnostic markers for various pathological conditions.

Exosomes play an important role in the viral life cycle. They help some viruses evade the immune response, thereby promoting the spread of viral infection. The roles of exosomes in the life cycles of such viruses as herpes simplex virus [9], human immunodeficiency virus [10], Epstein–Barr virus [11], and hepatitis A/C viruses [12,13] have been shown. Currently, the role of exosomes in the pathogenesis of influenza A virus (IAV) infection is also being actively investigated. Most works are devoted to the study of miRNAs, carried by the exosomes of infected cells, in host immune response regulation.

It has been shown that hsa-miR-1975, whose expression increases upon influenza A/WSN/33 (H1N1) infection of A549 cells, can be secreted into the extracellular space and transmitted to neighboring cells. Hsa-miR-1975, together with other proteins and nucleic acids, can promote interferon production and inhibit influenza virus replication [14]. Bronchoalveolar lavage fluid (BALF) studies of influenza virus-infected mice have revealed several miRNAs that may be involved in the innate immune response to viral infection. Small RNAs associated with the exosomes of BALF-infected mice (miR-483-3p, miR-374c-5p, miR-466i-5p, miR-203-3p) have been shown to significantly upregulate *IFN-β* expression, pro-inflammatory cytokine gene expression, and interferon-stimulated genes (ISGs), including *IL6*, *CCL2*, *TNF-α*, and *SP110*. The greatest upregulation of miR-483-3p was observed in macrophages and lymphocytes [15]. miR-483-3p-enriched exosomes derived from type II pneumocytes potentiated the expression of pro-inflammatory cytokine genes in vascular endothelial cells [16].

According to miRNA profiling of blood serum samples from IAV H1N1-infected patients, several miRNAs were detected; changes in their expression were also confirmed with IAV infection of A549 and MDCK cells [17]. The identified cluster of fourteen miRNAs act, among other things, as a regulator of *TGF-β* signaling and the *IL6* pathway. In addition, among the miRNAs of patients with IAV-induced acute respiratory distress syndrome (ARDS), miR-17-5p was found in exosomes. It has been shown that miR-17-5p highly reduces Mx1 expression in A549 cells, significantly enhancing viral replication [18].

An analysis of protein composition changes in extracellular vesicles has also been performed using human macrophages infected with influenza virus A/Udorn/72 (H3N2). It was shown that several important antiviral and pro-inflammatory cytokines (IFN-α1, IFN-α14, IFN-β, IL6, IL18, TNF-α) were increased in extracellular vesicles, resulting in the induction of a more effective antiviral response when transferred to neighboring cells. In addition, human copper metabolism MURR1 domain (COMMD) proteins, which are involved in NFκB inhibition, were found to be associated with extracellular vesicles from infected macrophages [19]. NFκB is known to be a central transcription factor that regulates the immune response and inflammation.

It has also been shown that EIF2 signaling, regulation of eIF4 and p70SK6 signaling, and mTOR signaling are highly upregulated in extracellular vesicles of IAV-infected macrophages. Thus, exosomes can help the virus evade the immune response and increase viral replication, as well as enhance the immune response to IAV infection.

The aim of this work was to study the composition of exosomes secreted by IAV-infected cells, as well as to study the functional role of these exosomes in the formation of the cellular environment in response to viral invasion. Exosomes were isolated from virus-infected A549 cells (EV) and from mock-infected A549 cells (E). The micro and mRNA compositions of these exosomes (E, EV) were compared. Gene expression patterns were compared between EV-stimulated A549 cells and E-stimulated A549 cells. The effect of EV on the ability of IAV to infect cells was also studied.

## 2. Materials and Methods

### 2.1. Cell Culture, Viruses, and Antibodies

Cell culture types A549 (human lung adenocarcinoma epithelial cells, ATCC CCL-185) and MDCK (Madin-Darby canine kidney cells, IRR FR-58) from the Smorodintsev Research Institute of Influenza (SRII) collection were used. Influenza virus A/Puerto Rico/8/1934 (H1N1) from the SRII collection was used to infect A549 cells. Mouse monoclonal antibodies to CD81 (Novus Biologicals, Denver, Colorado, USA) were used to identify exosome marker proteins. To detect influenza virus, mouse monoclonal antibodies to hemagglutinin (clone 10A12, SRII collection) were used. Peroxidase-labeled goat anti-mouse antibodies (GAM-HRP conjugate, Bio-Rad, Hercules, CA, USA) were used as secondary antibodies.

### 2.2. Exosome Production

The A549 cell line was used to produce exosomes. Cells were maintained in DMEM medium (Biolot, St. Petersburg, Russia) supplemented with 10% bovine serum (FBS, Biowest, Lakewood Ranch, FL, USA). Upon obtaining a 90% monolayer, cells were infected with influenza virus A/Puerto Rico/8/1934 (H1N1) at an infectious dose of 1 multiplicity of infection (moi) in serum-free medium (DMEM) supplemented with 1% antibiotic/antimycotic (Gibco, Grand Island, NY, USA). After one hour, supernatants (virus) were aspirated, and cells were washed twice with DPBS (Biolot, St. Petersburg, Russia). Maintenance medium (DMEM without serum) was then added, followed by incubation for 24 h (37 °C, 5% CO_2_). Culture media were collected and used to isolate exosomes. Mock-infected A549 cells were used to obtain control exosomes.

### 2.3. Next Generation Sequencing

Next generation sequencing (NGS) was used to compare the RNA compositions of exosomes released by IAV-infected (EV) cells and mock-infected (E) cells. Libraries for sequencing microRNA and RNA with a length of more than 200 nucleotides were obtained for E and EV using three biological replicates. Briefly, exosomes from cell supernatants were isolated with the exoEasy Maxi Kit (Qiagen, Hilden, Germany), followed by RNA extraction with the exoRNeasy Midi Kit. The QIAseq miRNA Library Kit and the QIAseq miRNA NGS 12 Index IL Adapter Kit (Qiagen, Hilden, Germany) were used for sample preparation and obtaining miRNA libraries. The NEBNext Ultra II Directional RNA Library Prep Kit for Illumina (NEB, Hitchin, UK) was used to obtain RNA libraries. NGS were performed on the HiSeq 2500 platform (Illumina, San Diego, CA, USA). The HiSeq Rapid SBS Kit v2 (Illumina, San Diego, CA, USA) and HiSeq Rapid SR Cluster Kit v2 (Illumina, San Diego, CA, USA) were used. The total concentration of pooled libraries applied to the cartridge was 10 pM.

The miRNA library sequence data were processed as follows. Reads were analyzed by FastQC, quantified, and analyzed by Qiagen GeneGlobe using the mirDB database. Sequence data for mRNA were analyzed using the FastQC [20] and Kraken2 [21] programs using the minikraken2_v1_8GB data package. The RSeQC [22], Qorts [23], and MultiQC [24] tools were used to assess data quality. Data was mapped to the human reference genome (GRCh38.p13) and annotated with coding regions using Gencode [25] and the STAR 2.7.9a software package [26]. To quantify the number of reads, the FeatureCounts software tool [27] was used. Read quantification was carried out for exon coding regions; overlapping regions of neighboring genes were not considered. Differentially expressed genes and expression statistics were also obtained using the DESeq2 function in the Phantasus 1.11.0 software package.

For functional comparative analysis of the data from the general list of genes obtained, a limit was used. Only those genes were considered whose differential expression ratios (fold change) were significantly greater than two: log_2_(change) ≥ 1 for upregulated genes; and log_2_(change) ≤ −1 for downregulated genes. Expression visualization was carried out using the Phantasus 1.11.0 software package [28]. Analysis of signaling and metabolic pathways activated under various conditions was performed using the MsigDB (http://software.broadinstitute.org/gsea/msigdb, accessed on 1 December 2021) and Reactome (https://reactome.org, accessed on 1 December 2021) databases [29]. Pathway data were obtained from the GO [30], MsigDBHallmarks [31], and Reactome [32] databases. The FGSEA tool [33] was also used.

### 2.4. Isolation of Exosomes and Influenza A Viral Particles

In the first step, concentrated exosome samples were obtained from cell media by the ultracentrifugation method described [34]. Media were centrifuged at 3000× *g* for 30 min (4 °C) to remove cells and their debris, followed by collection of supernatants for further processing. Supernatants were centrifuged at 10,000× *g* for 30 min (4 °C) to remove large vesicles; pellets were discarded. Exosomes were concentrated from supernatants at 110,000× *g* for 2 h (4 °C) using the Optima XPN ultracentrifuge (Beckman Coulter, Brea, CA, USA). Pellets were resuspended in PBS.

In the second step, concentrated exosomes (350 µL obtained after ultracentrifugation) were applied to a sucrose gradient to separate exosomes and influenza viral particles. A sucrose step gradient (55%, 45%, 35%, 25%, 15%) in PBS/D_2_O was used, with higher concentrations at the bottom. The volume of each fraction was 930 µL. Ultracentrifugation was performed in an Optima XPN centrifuge (Beckman Coulter, Brea, CA, USA) at 210,000× *g* for 20 h. The sucrose gradient was then collected as 20 fractions (250 µL each) and examined by dot blotting to identify exosomal and viral protein markers. Fractions containing exosomal protein markers were pooled, diluted in PBS to 50 mL, and centrifuged at 110,000× *g* for 2 h (4 °C). Pellets were resuspended in 100 µL of PBS.

### 2.5. Dot Blotting

A supported nitrocellulose membrane (Bio-Rad, Hercules, CA, USA) was first soaked in distilled water, followed by PBS, for 10 s. Samples were applied to dried membrane in a volume of 2 µL. After complete drying of the membrane, it was incubated in a PBST (PBS with 0.1% Tween-20) blocking solution containing 5% Blotting Grade Blocker Non-Fat Dry Milk (Bio-Rad, Hercules, CA, USA) for 1 h, followed by a PBST wash for 5 min. The membrane was then incubated in primary antibody solution (diluted in blocking solution to 1 µg/mL) for 1 h. The membrane was washed in PBST (15 min twice) and then incubated with labelled secondary antibodies (diluted in blocking solution to 1 µg/mL) for 1 h. The secondary antibodies were goat anti-mouse horseradish peroxidase conjugates (Bio-Rad, Hercules, CA, USA). The membrane was washed with PBST (10 min twice). Results were visualized using the Clarity Western ECL Substrate kit (Bio-Rad, Hercules, CA, USA) and the ChemiDoc XRS+ imaging station.

### 2.6. Transmission Electron Microscopy

Samples for electron microscopy were prepared according to a negative staining protocol. Samples (20 µL) were applied to Parafilm M film (Pecheney Plastics Packing, Shelbyville, TN, USA). A copper, collodion-coated, 300 mesh microscopy grid (Electron Microscopy Science, Hatfield, PA, USA) was then placed over the sample and incubated for 20 s. The sample was then removed, and the mesh was washed twice with 20 µL of distilled water for 20 s. Water was removed, and the preparation was contrasted with a 1.5% aqueous solution of phosphotungstic acid sodium salt for 20 s. The contrast solution was removed, and the mesh was dried at room temperature. Specimens were examined using a JEM 1100 electron microscope (JEOL, Tokyo, Japan) with an accelerating voltage of 80 kV.

### 2.7. Nano-Tracking Analysis

Nano-tracking analysis (NTA) was used for the analysis of particle size distribution in liquids based on correlation of Brownian motion with single-particle hydrodynamic diameter. Size distribution and concentration were analyzed with the NanoSight LM10 device (Malvern Panalytical, Malvern, UK) using a cuvette with a 405 nm laser (Nano-Sight, Malvern Panalytical, Malvern, UK). To obtain better measurements, suspensions were diluted up to 10,000-fold to achieve optimal concentrations. Measurements were performed in triplicate with the following parameters for all analyses: 60 s video captures with camera level 16; lowest expectable particle size = 30 nm; and detection threshold = 7. Video processing, size/concentration analysis, and statistical evaluations were carried out using NanoSight NTA 2.3 software (Malvern Panalytical, Malvern, UK).

### 2.8. Determination of Infectious Activity

The presence of infectious viral particles in exosome samples was determined by endpoint dilution assay using MDCK cells. Ten-fold dilutions of positive controls (influenza A/Puerto Rico/8/1934, H1N1) and negative controls (DPBS), as well as exosome samples, were prepared in DMEM culture medium (Gibco, Grand Island, New York, USA) with added 2.5 µg/mL TPCK-trypsin (Sigma-Aldrich, St. Louis, MO, USA) and 1% antibiotic/antimycotic (Gibco, Grand Island, NY, USA). To determine TCID_50_ (50% tissue culture infectious dose), the obtained samples (100 µL) were added to a one-day monolayer of MDCK cells cultivated in 96-well plates (Nunc, Roskilde, Denmark) and incubated for 72 h (37 °C, 5% CO_2_). Hemagglutination reactions were then performed to evaluate the results.

For hemagglutination reactions, 50 µL of sample was transferred into the wells of a 96-well plate and 50 µL of a 0.5% suspension of chicken erythrocytes was added. The plate was incubated at 4 °C for 30 min, and the presence or absence of erythrocyte agglutination was recorded. Calculation of the TCID_50_ was carried out according to the method of Reed and Muench [35] and expressed in lgTCID_50_/mL.

### 2.9. Stimulation of A549 Cells by Exosomes

The biological activities of the isolated E and EV samples were verified by RT-PCR using A549 cells cultured at 37 °C with 5% CO_2_. A549 cells were seeded at 2 × 10^5^ cells per well in 12-well plates (Nunc, Roskilde, Denmark). After 24 h, the monolayer was washed twice with DPBS, and exosomes were added at a concentration of 10^5^ particles/well in 0.3 mL DMEM culture media (Gibco, Grand Island, NY, USA). Three wells were filled with 0.3 mL of mock DMEM medium to serve as cell controls. After incubation for one hour, DMEM containing 1% Antibiotic-Antimycotic (Gibco, Grand Island, NY, USA) was added to the cells to a final volume of 1 mL. At 24 h after stimulation, total RNA was extracted from cells using Trizol (Invitrogen, Waltham, MA, USA) according to the manufacturer’s instructions.

### 2.10. Real-Time Quantitative PCR

After the generation of cDNA by reverse transcription, including preliminary DNAse treatment of samples, mRNA levels of specific genes were quantified by real-time PCR (RT-qPCR). These were *RIG1*, *IFIT1*, *MDA5*, *MxA*, *PKR*, *SOCS1*, *COX2*, *AnxA1*, *NFκB*, *IL6*, and *IL18*. Relative expression was calculated by the ΔΔCt method [36] using GAPDH as the normalization gene. All determinations were performed in triplicate. The primer and TaqMan probe sequences are supplied in Table 1.

To identify IAV genomic components in exosomes, reverse transcription was performed using dT16 or random octamer primers, followed by PCR using primers that specifically detect the *NS*, *M*, and *PA* viral genes. Real-time PCR was performed using HS-qPCR SYBR Blue (2×) (Biolabmix, Novosibirsk, Russia) for the *NS*, and *PA* genes. Conditions were 40 cycles of 95 °C denaturation, 58 °C (55 °C for PA) annealing, and 72 °C extension. For the *M2* gene, real-time PCR was performed using HS-Taq PCR (2×) (Biolabmix, Novosibirsk, Russia).

### 2.11. Infection of Cells with Influenza A Virus in the Presence of Exosomes

MDCK cells (2 × 10^4^ cells/well) were seeded overnight into 96-well plates before ‘preventive/therapeutic’ treatment with the indicated exosomes. For treatment, cells were washed in DPBS, and the medium was replaced with serum-free AlphaMEM (Biolot, St. Petersburg, Russia) with E, EV (about 1.3 × 10^8^ particles per well) or without exosomes (‘mock-treated’). After 24 h of stimulation, exosome-containing supernatants were removed, and cells were infected with A/California/07/09 (H1N1pdm09) at 10^−1^–10^−7^ dilution (corresponding to a dose from 50000 to 0.05 TCID_50_ per well). After 1 h of virus adsorption, inoculum was removed, and exosome-containing supernatants were returned to the cells. For multicycle IAV replication, the medium after infection additionally contained TPCK-treated trypsin (Thermo Fisher Scientific, Waltham, MA, USA) at a final concentration of 1.25 μg/mL. Viral infectious activity in one-cycle IAV replication (without trypsin) and multicycle IAV replication was measured at 20 or 48 h post-infection.

The level of viral replication was measured in supernatants using real-time PCR with the *M2* gene as described in Section 2.10. It was also indirectly measured using In-Cell ELISA with anti-NP antibodies as follows. Cells in monolayer were fixed with cold 80% acetone. After washing with a PBST solution and blocking with 5% dried milk (Bio-Rad, Hercules, CA, USA), 100 µL of monoclonal antibody 4H1 (form the collection of the Smorodintsev Research Institute of Influenza), which is specific to IAV NP, was added to the wells (1 µg/mL). The binding of monoclonal antibodies was detected using GAM-HRP (Bio-Rad, Hercules, CA, USA). Peroxidase reaction was performed using the TMB Peroxidase EIA Substrate Kit (Bio-Rad, Hercules, CA, USA). Reactions were stopped by the addition of 100 µL of 1 N H_2_SO_4_ to each well. Optical densities were measured as the difference between OD_450_ and OD_650_ on a CLARIOstar microplate reader (BMG LABTECH, Ortenberg, Germany). The data were processed using GraphPad Prism.

## 3. Results and Discussion

### 3.1. Exosomal miRNA and mRNA Profile Changes with Influenza Virus Infection

**Figure 1:**
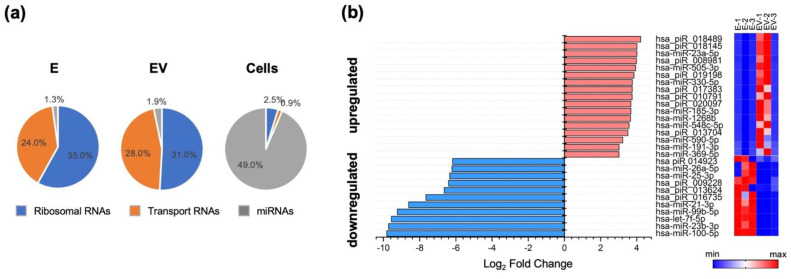
(**a**) Different types of RNA levels in exosomes samples (E—exosomes from mock-infected cells; EV—infected-cell-secreted exosomes), compared to control cells (‘Cells’). For the three pie charts, the unshown percentages adding up to 100% (39.7%, 39.1%, 47.6%) correspond to meaningless reads (no adapter, too short, UMI defective reads, uncharacterized sequences). (**b**) MicroRNAs upregulated (log_2_ fold change > 1) and downregulated (log_2_ fold change < 1) after influenza A infection of A549 cells. Heat maps present the level of miRNA expression: E-1, E-2 and E-3—three independent samples of exosomes from mock-infected cells; EV-1, EV-2 and EV-3—three independent samples of infected-cell-secreted exosomes.

In the first stage, we assessed how the level of exosomal microRNA changed in response to IAV infection. The composition of microRNAs isolated from IAV-infected A549 cell exosomes (EV) differed from that of microRNAs isolated from mock-infected cells (E). Exosomes, both E and EV, contained increased amounts of ribosomal and transport RNAs, alongside significantly lower amounts of miRNAs, compared to control cells (Figure 1a).

When comparing E and EV miRNAs pools, we found that miR-100-5p, miR-23b-3p, let-7f-5p, miR-99-5p, miR-21-3p, miR-26a-5p, miR-25-3p, and some piRNAs were downregulated in response to IAV stimulation of cells (Figure 1b). The amounts of these small RNAs in EV were 64 to 800-fold lower than in E. Influenza infection also led to a slight (14-fold) increase in such exosomal microRNAs as miR-23a-5p, miR-505-3p, miR-330-5p, miR-1268b, and miR-185-3p (Figure 1b).

Among the downregulated miRNAs, hsa-miR-21-3p and hsa-miR-26a-5p should be highlighted. It has been shown that hsa-miR-21-3p can promote influenza virus replication by suppressing expression of histone deacetylase 8. Thus, downregulation of miR-21-3p can act as a cell defense mechanism during viral infection [37]. It has also been shown that miR-26a-5p activates the type I interferon signaling pathway and promotes the production of IFN-stimulated genes (ISGs), leading to suppression of viral replication. miR-26a-5p also acts on ubiquitin-specific protease 3, which is a negative regulator of the type I IFN pathway [38]. Thus, downregulation of miR-26a-5p, on the contrary, can promote influenza virus replication.

Among upregulated miRNAs in EV, hsa-miR-23a-5p and hsa-miR-548c-5p should be noted. Hsa-miR-23a-5p inhibits autophagy activation via the TLR2/MyD88/NFκB pathway by acting on *TLR2* [39], thus promoting the spread of a bacterial pathogen. Upregulation of the miR-548 family (including miR-548c-5p) promoted cell infection by enterovirus-71 and vesicular stomatitis virus. Thus, miR-548 regulates the host antiviral response via direct targeting of *IFN-λ1*, by reducing *IFN-λ1* expression [40].

The identified miRNAs may be involved in the regulation of the cell’s immune response to viral infection. Their effects may contribute to cell protection (miR-21-3p). They may also enhance pathogen replication or suppress the immune response (miR-26a-5p, miR-23a- 5p, miR-548c-5p). In the published review of Zheng et al. [41], devoted to the role of miRNAs in IAV infection, the function of miRNA as modulators of the immune response was also highlighted, and two miRNAs (miR-21-3p and miR-26a-5p) were reported. At the same time, we found in exosomes some new miRNAs which function as immune response regulators during microbial or viral infection according to published descriptions. Thus, our results are in agreement with the current understanding of the role of miRNAs in IAV infection development and could shed light on the mechanisms of miRNA transmission.

**Figure 2:**
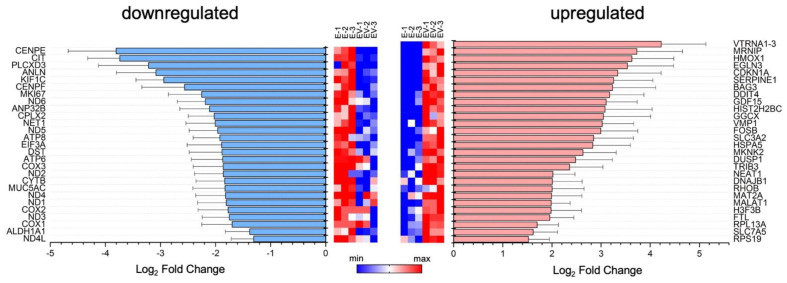
List of mRNAs with different levels between exosomes from infected and mock-infected cells. Significantly downregulated (log_2_ fold change < −1) and upregulated (log_2_ fold change > 1) genes are shown. On the left and the right sides of the figure, the names of downregulated and upregulated genes are presented. Heat maps reflect the level of mRNA expression: E-1, E-2 and E-3—three independent samples of exosomes from mock-infected cells; EV-1, EV-2 and EV-3—three independent samples of infected-cell-secreted exosomes.

In the next stage, the content of exosomes was studied by comparing RNA pools with a length of more than 200 nucleotides (mRNA, long non-coding RNA, immature RNA, etc.), isolated from E and EV. Surprisingly, most of the reads (about 75%) were for long, non-coding RNAs. About 20% were for intergenic regions, and less than 10% were predominantly for protein-coding regions. When comparing the two groups (E, EV), about 1,100 differentially expressed genes were identified. Next, we selected only those genes whose differential expression ratios were significantly greater than two. Lists of such upregulated (log_2_ fold change ≥ 1) and downregulated (log_2_ fold change ≤ −1) genes are presented in Figure 2.

According to the Reactome and GSEA databases, we tried to categorize genes identified in exosomes (p_adj_ < 0.05) into groups based on shared function, involvement in a metabolic pathway, presence in a specific cellular location, or other categorizations, e.g., functional pathways. However, we were unable to identify with statistical reliability genes involvement in any pathway, or grouping of genes together, based on similarity of function. We considered it plausible that the set of differentially expressed genes in EV may be distributed randomly. As such, we tried to determine how individual mRNAs found could influence the cellular environment.

Among the downregulated mRNAs, we observed genes encoding proteins of the mitochondrial electron transport chain (i.e., some subunits of NADH, ATP synthase, cytochrome C oxidase subunit 1, subunit 2, subunit 3). In addition, expression of the *MUC5AC* gene (encoding the glycoprotein Mucin-5AC) was reduced in EV. Mucin-5AC lines the epithelium of the respiratory tract and protects cells from inhaled microorganisms [42].

Among the upregulated mRNAs, several genes encoding proteins associated with the cell’s immune response were distinguished: *SLC7A5*, *SLC3A2*, *RPL13A*, *MKNK2*, and *TRIB3*. The role of the SLC3A2 and SLC7A5 complex in the case of hepatitis C viral infection has been shown [43]. The virus increases the concentration of the complex, which leads to the activation of mTORC1 signaling. Thus, the SLC3A2/SLC7A5 complex plays an important role in the penetration of the virus into the cell [43]. The 60S ribosomal protein L13a, encoded by the *RPL13A* gene, is a component of the gamma interferon-activated inhibitor of translation complex, which mediates interferon-gamma-induced transcript-selective translation inhibition in inflammatory processes [44]. MAP kinase signal-integrating kinases (MNKs) play a key role in positive regulation of the synthesis of the inflammatory cytokine TNF-α. They may also be involved in the regulation of other cytokines [45]. It has also been shown that MNKs are important elements in the control of IFN-γ-inducible interferon-stimulated gene mRNA translation [46]. *TRIB3* gene expression is induced under stressful conditions: endoplasmic reticulum stress, nutrient imbalances, oxidative stress, hypoxia, or viral infection [47]. TRIB3 is a negative regulator of NFκB levels both using a negative feedback mechanism, and acting as a downstream effector of the ATF4-CHOP pathway [47,48,49,50]. NFκB activation is associated with the development of an antiviral response of the cell and the induction of type I IFN. During viral infection, activation of NFκB-dependent signaling occurs, and induction of cytokines and chemokines (IL6, IL8, TNF-α, IFN-β) at the mRNA level is noted [51,52].

Both mRNAs and miRNAs could be delivered and released into recipient cells. Moreover, transferred exosomal mRNA can be translated after entering, forming functional proteins [53,54,55]. It should be noted that the changes in the mRNA profile of exosomes during influenza infection can be associated with both the regulatory function of RNAs transferred to neighboring cells, and processes occurring in infected cells.

Thus, we observed that the level of *TRIB3* (a negative regulator of NFκB), as well as that of other genes involved in viral replication, increased in EV. This may indicate suppression of pro-inflammatory signaling cascades. Based on literature data on the role of miRNAs in secreted exosomes (IAV-infected cells, patient isolates), as well as based on our data on miRNA/mRNA EV content, we decided to further study the activity of EV samples. By examining their influence on the expression of genes associated with host immune response, the goal was to clarify the impact of IAV infection on the cellular microenvironment.

### 3.2. Isolation of Exosomes Secreted by IAV-Infected A549 Cells, Free of Viral Particles

A critical step in studying the effect of EV on the cellular microenvironment was obtaining exosome samples that do not contain viral particles. It should be noted that the physicochemical properties of exosomes and IAV virions are similar (such as floating density, size, presence of host proteins in the particles [56,57]). Furthermore, the duration of one IAV replication cycle is 8 h on average, while most protocols depend on exosome accumulation in cell medium for at least 24 h [34]. Thus, it cannot be ruled out that exosomes secreted by IAV-infected cells (isolated and analyzed in the previous stage) may also contain viral particles. Even though there are no clear data in the literature on the presence of host miRNAs or mRNAs in the virion structure, for further experiments of studying the effect of EV on intact A549 cells, it was necessary to obtain purified exosomes free from viral particles. Several of the standard exosome isolation methods have drawbacks that make them unsuitable for this goal. These include straightforward sedimentation (cell medium ultracentrifugation), size-based methods (gel filtration), and surface protein-based methods (affinity chromatography with antibodies to exosomal surface markers). Thus, we needed to develop a method for separation of exosomes from IAV particles.

One exosome isolation method uses a sucrose gradient, allowing exosomes to be separated by their floating density. The floating density of exosomes is 1.15–1.19 g/mL [34], while that of the influenza virus is from 1.18 g/mL or more (up to 1.26 g/mL [58]). Their floating densities partially overlap. However, it was decided to use a sucrose gradient to separate exosomes and virions, while using in further analysis only exosomes from those fractions that should not be contaminated with viral particles. Based on the floating density of exosomes, we expected exosomes to be located in the upper sucrose gradient fractions (lower density) and influenza virions to be located in the lower fractions (higher density).

A549 cells were infected or mock-infected with influenza A/Puerto Rico/8/1934 (H1N1) virus at the dose of 1 moi without trypsin. Thus, no cell death was achieved after 24 h of incubation because we observe one-cycle of infection. After exosome production, culture medium purification, and sedimentation preparation (exosome or exosomes-and-virus), samples were loaded onto a sucrose gradient and centrifuged overnight to separate exosomes and virions (see Section 2.4). After centrifugation, the gradient was collected as 20 fractions and analyzed for the presence of viral and exosomal proteins.

**Figure 3:**
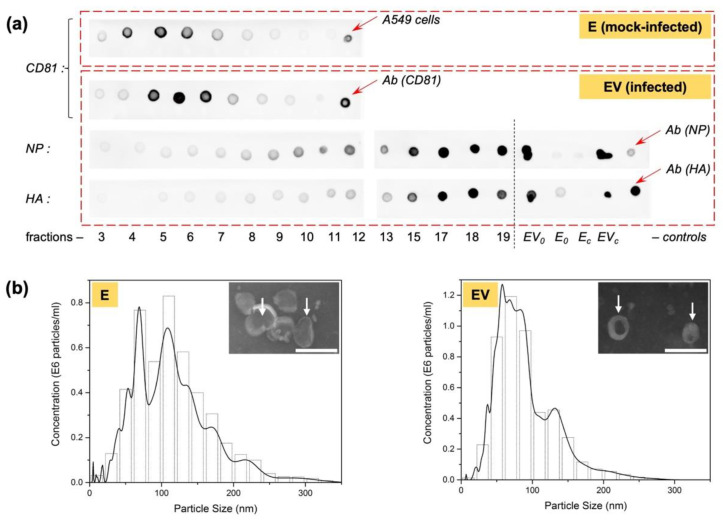
Exosome sample analyses. (**a**) Sucrose gradient fraction (from 3 to 19) dot blot with antibodies to exosome marker protein (CD81) and to IAV structural proteins (nucleoprotein, NP, and haemagglutinin, HA). Positive controls of mock-infected A549 cells, as well as pure antibodies, Ab(CD81), Ab(NP), and Ab(HA), are marked by red arrows. As controls, infected-cell-secreted exosomes before sucrose gradient (EV_0_) and infected cells (EV_c_) were also tested. To check unspecific binding of antibodies to NP and HA used with exosomes from mock-infected cells, exosomes from mock-infected cells before sucrose gradient (E_0_) and mock-infected cells (E_c_) were also tested. (**b**) NTA analysis (left is E exosomes, diameter 116 ± 53 nm; right is EV exosomes, diameter 90 ± 41 nm); and (**b,** inset) transmission electron microscopy of pooled fractions (scale bar is 200 nm; white arrows mark some exosomes).

Figure 3a shows the results of a dot blot of the fractions with the exosomal marker protein CD81. The highest CD81 content fell in fractions 4 to 6 in mock-infected exosomes (E) and in fractions 5 to 7 in exosomes from infected cells (EV); this indicates the presence of exosomes mainly in these fractions. At the same time, when analyzing EV fractions for IAV structural proteins (nucleoprotein, hemagglutinin) (Figure 3a), it was shown that hemagglutinin (HA) was clearly visualized in fractions 9–10, with its concentration increasing with increasing fraction number (i.e., with increasing sucrose density). Nucleoprotein (NP) was barely noticeable in fractions 3 to 7. From fraction 8, its concentration began to increase. The presence of hemagglutinin could indicate both weak unspecific binding, and the presence of full-fledged viral particles, which were observed mainly in the lower fractions and did not intersect with the gradient’s exosomal region. The presence of a small amount of nucleoprotein in the fractions containing exosomes may be associated with the accidental capture of this protein during exosome formation, since it is one of the most actively synthesized proteins during IAV infection.

For pooled samples E (fractions 4, 5, 6) and EV (fractions 5, 6, 7), electron micrographs were also obtained (Figure 3b, insets). The observed particles corresponded to exosomes in shape (cup-shaped) and size (about 100 nm).

To confirm the absence of active, infectious IAV particles in the purified exosome samples, we measured the viral infectious activity by titration in MDCK cells, followed by viral hemagglutination assays to detect viral particles. The absence of any chicken erythrocyte agglutination in all dilutions of EV (after purification by sucrose gradient) was shown. Both the positive control of the virus and the medium from infected cells (before purification) caused erythrocyte agglutination (viral titers were 2.0 ± 0.5 lgTCID_50_/mL and 1.5 ± 0.5 lgTCID_50_/mL, respectively). Thus, by the sucrose gradient procedure, exosomes secreted by IAV-infected cells, free from viral particles, were obtained.

Three identical experiments were carried out to obtain mock-infected exosomes and exosomes from infected cells. Because of the low quantity of exosomes produced by adherent A549 cells, all three biological replicates were pooled for further experimental analysis of activity, resulting in the formation of two samples, E (exosomes form mock-infected cells) and EV (exosomes from virus-infected cells). These were characterized by NTA (Figure 3b). Using NTA, particle with size characteristic of exosomes (approximately 100 nm) were also observed. Sample exosome content was also estimated, which made it possible to equalize their concentration to 0.7 × 10^11^ particles/mL for further study of their effect on intact cells.

### 3.3. The Effect of E and EV on Intact A549 Cells

**Figure 4:**
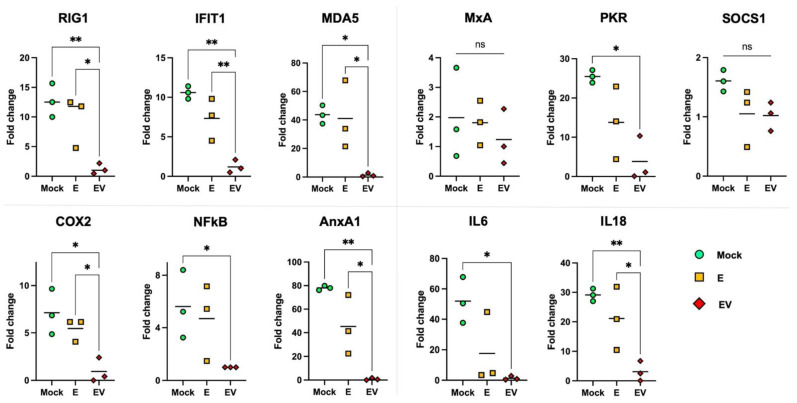
Expression profile changes in E- and EV-treated cells. Three separate stimulations of cells (by pooled E and EV samples) were performed. Data are presented as measured points and mean value. ‘Mock’ (control cells)—mock-treated A549 cells. All fold changes were calculated considering the mean gene expression in EV as 1. Statistical analysis was performed using one-way ANOVA: **—*p* < 0.01; *—*p* < 0.05, ‘ns’—*p* > 0.05 (not significant).

To evaluate the effect of exosomes on intact cells, A549 cells were stimulated with purified samples of E or EV. After 24 h of stimulation, we assessed the mRNA levels of specific genes (participants in the immune response, cytosolic sensors, the transcription factor NFκB) using RT-PCR (Figure 4) to examine potential expression changes. We showed that expression of *COX2* and *NFκB* genes (key regulators of the inflammatory process and the immune response [51,59,60]) decreased in cells into which EV exosomes were introduced. It is important to note that E exosomes (obtained from mock-infected A549 cells) did not affect *COX2* or *NFκB* mRNA levels.

Since activation of COX2 and NFκB induces TNF-α, IFN-β and IL6 [61,62], we tested whether inhibition of *COX2* and *NFκB* expression would lead to a decrease in the level of these cytokines. According to the results, *IL6* mRNA was at an undetectable low level after cells were treated with EV exosomes. Expression of *IL18* (IFN-γ-induced factor), which is constitutively expressed in epithelial cells, also dropped significantly with EV treatment [63].

The innate immune response requires IFN induction and activation of interferon-stimulated genes (ISGs) [64]. Estimation of the expression levels of the most important ISGs showed that the introduction of EV exosomes led to a decrease in the levels of *RIG1*, *MDA5*, *PKR* and *IFIT1*, but not of the canonical antiviral gene *MxA*. No changes were found in the mRNA level of the *SOCS1* gene, a negative regulator of JAK/STAT signaling cascades [65], either. The fact that the key “marker” of viral infection *MxA* [64], as well as *SOCS1* (important in regulation of cytokine signaling), did not change their expression in response to E or EV treatment, further supports an absence of influenza virions in the isolated exosome samples.

There is a close relationship between the cytosolic RNA sensors RIG1, MDA5 and IFIT1, which forms a unique system of cellular protection from foreign RNAs [66]. Annexin A1 (AnxA1) is able to enhance the expression of *RIG1* and type I IFN, but its activity is associated with apoptosis during influenza infection. Conversely, knockout of the *AnxA1* gene leads to a decrease in the expression of *RIG1*, type I *IFN*, and *IFIT1* [67].

It was suggested by Ge et al. [68] that the exosomal pathway might participate in the process of influenza virus infection, and blocking the secretion of exosomes reduced viral RNA replication. Moreover, more than 30 genes related to interferon activity have been found that are potential targets of influenza-stimulated exosomal miRNAs, including *IFNA*, *IFNAR1*, *OAS*, *OASL*, *IRF*, *JAK1*, and *STAT1*. All these genes were overexpressed, while the expression of *SOCS1* was suppressed by miRNA hsa-let-7e. The miRNAs also upregulated the P53 signaling pathway, MAPK signaling pathway, and the TNF and apoptosis signaling pathway [68]. Surprisingly, our results show another miRNA profile in exosomes (Figure 1). At the same time, we noted lowered expression of some ISGs after treatment of naïve cells with exosomes (Figure 4), while *SOCS1* mRNA levels did not change. In Ge’s work [68], IAV strain A/H1N1pdm09 was used. We infected A549 cells with A/Puerto Rico/8/1934 (H1N1). It is well known that the ability to evade the host immune response can vary greatly between IAV strains. For instance, IAV can use PA-X and NS proteins to suppress host cell protein translation [69,70]. PA-X is a major contributor in reducing general host protein expression in virus-infected cells; while NS1 shutoff activity specifically targets host mRNAs related to IFN signaling pathways and cytokine release [69]. Most human IAV strains have low PA-X shutoff activity, but the 2009 pandemic H1N1, on the contrary, utilized this mechanism [70]. Consequently, the ability of IAV to suppress the innate immune response and block IFN production is strain-specific [70], which may explain some discrepancy in the results.

Thus, according to our results, we can conclude that introduction of EV specifically suppressed the recognition system of exogenous viral RNAs. On the other hand, the simultaneous decrease in the expression of the *RIG1*, *MDA5*, *IFIT1*, and *AnxA1* genes can be considered as evidence of suppression of the pro-apoptotic signaling cascade. The latter is in good agreement with data on a decrease in *NFκB* expression, as well as NGS data on an increased level of *TRIB3* mRNA.

### 3.4. Identification of Viral RNA in Exosomes

All the results obtained in this work indicate a pronounced immunosuppressive effect of exosomes secreted by infected cells on intact A549 cells (Section 3.2). The secretome of infected cells (Section 3.1) also contains some cellular factors that can potentially cause the same effect. In addition, the fact that not only proteins and RNA of the host cell, but also proteins or RNA of the influenza virus can also be transferred from an infected to a healthy cell cannot be excluded.

A key IAV protein, which has an immunomodulatory effect, is NS1. Through NS1, influenza viruses can limit interferon signaling at various levels, antagonize ISGs, and thereby promote influenza virus replication. TLR and RLR pathways are the main pathways in the type I interferon reaction induced by the influenza A virus. It has been shown that NS1 inhibited the activation of IRF3 and NFκB. In virus-infected cells, NS1 can interact with various host proteins to suppress cellular mRNAs through RIG receptor interactions, as well as competitive binding of dsRNA. NS1 can also interact with various ISGs (protein kinase R, OAS, interferon stimulating gene 15, zinc finger antiviral protein) to antagonize the host’s natural immune response [71]. At the same time, the presence of the NS1 protein in exosomes was indirectly shown earlier [57]. To perform its function, a sufficiently high concentration of exogenous NS1 in the extracellular environment is required, or it must act by inducing a conformational transition, as was previously suggested [72], but the transfer of NS1 from an infected cell to a healthy cell in the form of mRNA is not excluded either.

Using PCR with a preliminary stage of reverse transcription (RT), we assessed the presence of specific components of the viral genome in the exosomal cargo. To be able to identify products derived from IAV mRNA, RT was performed with both dT16 and Random9 primers. The use of dT16 primers (for hybridization with polyA tails) permits the detection of only mRNA encoding the studied proteins (GAPDH, M2, NS and PA), while the use of Random9 primers permits detection of mRNA, cRNA and genomic RNA simultaneously. According to the results obtained (Table 2), both exosome samples E and EV contained approximately the same small amount of mRNA of the *GAPDH* ‘housekeeping’ gene. In addition, exosome E, as expected, did not contain components of the viral genome. PCR showed the presence of the viral genes *M2*, *NS* and *PA* in EV. At the same time, *M2* and *NS* could be present either as mRNA or any other form of viral RNA because of the difference in the cycles received using dT16 and Random9 primers. The absence of *PA* mRNA in EV (the absence of any product using dT16 primes) may be explained both by the low copy number of this gene and, presumably, by a more specific selection of viral genes into exosomes. However, the question of viral mRNA content in EV is not fully understood and requires further study.

### 3.5. Effect of Exosomes on Influenza A Virus Replication

**Figure 5:**
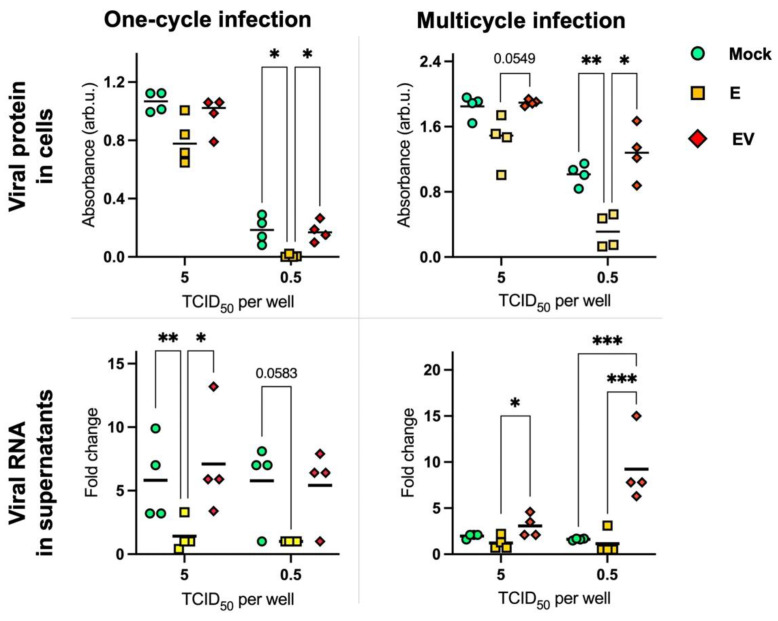
Viral protein (nucleoprotein) levels in cells and viral RNA in supernatants after IAV infection of A459 cells (treated with E, EV, or mock-treated) 20 h post-infection without trypsin (one-cycle-infection) and 48 h post-infection with trypsin (multicycle infection). The statistical analysis was performed using two-way ANOVA: ***—*p* < 0.001; **—*p* < 0.01; *—*p* < 0.05. *p* values of 0.0549 and 0.0583 (not significant) are marked. To calculate fold change, the mean gene expression in E was considered as 1.

To analyze the direct influence of infected-cell-secreted exosomes on influenza A viral replication, we infected MDCK cells, which were mock-treated or treated (by E or EV), with IAV at different doses (10-fold viral dilutions from 5 × 10^4^ to 5 × 10^−2^ TCID_50_ per well). Significant differences were obtained using a dose of 5 and 0.5 TCID_50_ per well (Figure 5). To quantify the level of viral proteins in infected cells, we performed In-Cell ELISA with antibodies to viral NP (Figure 5, row ‘viral protein in cells’). To analyze the IAV quantity in the supernatants, we performed PCR with primers to the viral segment *M* (Figure 5, row ‘viral RNA in supernatants’).

We observed that the level of viral nucleoprotein was significantly lower in E-treated cells in relation to EV-treated or mock-treated cells using the dose 0.5 TCID_50_/well in both one-cycle and multicycle infection. At the same time, IAV levels in supernatants were significantly lower in E-treated cells using the dose 5 TCID_50_/well. It should be noted separately the situation detected during multicycle infection in supernatants using the dose 0.5 TCID_50_/well: the IAV level was significantly higher in EV-treated cells.

Based on our data that EV has an immunosuppressive effect on naïve A549 cells, we could expect that after EV treatment IAV replication would be more effective than in mock-treated cells. We observe such a situation in supernatants after several cycles of viral replication at a dose of 0.5 TCID_50_ per well (Figure 5, lower right corner.

We hypothesize that the same level of viral replication in mock-treated and EV-treated cells in the other experimental conditions is related to the presence of ‘natural’ infected-cell-secreted exosomes in the influenza A virus stock solution which was used for cell infection. The presence of exosomes in ‘purified’ IAV samples has been shown earlier by Hutchinson et al. [57]. Thus, EV-treated and mock-treated cells may receive a similar influence from infected-cell-secreted exosomes, although of different origin.

At the same time, by addition of exosomes from mock-infected cells (E), we make the influence of infected-cell-secreted exosomes in the IAV sample lower. Further, E exosomes could transmit signals which provoke an immune response, interfering with unhindered viral replication. During our NGS study, we found a microRNA (miR-26a-5p) that normally should stimulate ISGs, which was upregulated in E exosomes and downregulated in EV exosomes.

Thus, we observed a two-sided effect of exosomes during IAV infection. On one hand, EV exosomes make more effective viral replication, which was reliably shown at low doses by an increase in the virus concentration in supernatants. On the other hand, E exosomes stimulate an adequate response of cells to viral infection, resulting in decreased viral replication. It should be noted that we did not observe earlier the ‘stimulative’ effect of E exosomes (Figure 4); this could be related to the presence of natural exosomes in cell supernatants of mock-treated cells, secreted by naïve cells during the experiment. However, an entire series of experiments would be needed to clarify the direct mechanisms of exosome (E, EV) influence on IAV replication and cellular responses. These will likely be the scope of further studies.

The protective role of exosomes secreted during influenza infection has been shown earlier. It is related with direct interaction of exosomal α2,3 and α2,6-linked sialic acids with influenza virions, leading to the inactivation of the latter [73,74]. In our study, we did not mixed IAV with exosomes: we collect exosomes, when infecting the cells, and return them after removal of the virus. Thus, in our scheme, exosomes could not interact with IAV particles directly. As a result, other mechanisms of IAV inhibition may also exist, suggesting the exosomal pathway might participate in the process of influenza virus infection.

It should also be noted that Bedford et al. [73] showed the antiviral activity of exosomes secreted in mouse lungs, which contained not only epithelium cell RNAs. Since immune cells (macrophages, dendritic cells) make a decisive contribution to cytokine secretion, a direct comparison of our results with the results obtained in mice cannot be carried out. Exosomes derived from influenza virus-infected mice can contain viral RNA segments or proteins which can trigger the innate immune response, mediated by excessive cytokine and chemokine production. A549 cells, which were used in our work, are not ‘professional’ antigen-presenting cells. However, they have their own cytokine expression profile [73]. In our work, it turned out that intact-cell exosomes did not influence expression of RNA sensors, ISGs, or cytokines. Meanwhile, EV exosomes suppressed the innate immune response.

## 4. Conclusions

We have shown that exosomes secreted by influenza-virus-infected cells are involved in the regulation of the immune response of neighboring uninfected cells. We noted changes in exosomal composition that are likely involved in host immune response regulation. Furthermore, addition of infected-cell-secreted exosomes completely suppressed, or significantly reduced, the expression in uninfected cells of such genes as *RIG1*, *IFIT1*, *MDA5*, *PKR*, *COX2*, *NFκB*, *AnxA1*, *IL6* and *IL18*. This indicates that these exosomes exert an immunosuppressive effect. Such activity could potentially be mediated in two ways: inclusion of host factors or inclusion of viral factors. Changes in cellular miRNA/mRNA composition in exosomes were seen that relate to the regulation of the immune response during influenza infection. Examples include both upregulation (miR-23a-5p, miR-548c-5p, *RPL13A*, *MKNK2*, *TRIB3*) and downregulation (miR-26a-5p). The presence of viral mRNAs in infected-cell-secreted exosomes (i.e., mRNA encoding the immunosuppressive protein NS1 of the influenza virus) may also influence the gene expression observed. Treatment of A549 cells with E and EV exosomes, followed by IAV infection, showed the protective activity of E relative to EV exosomes: molecular products of viral replication were higher in cells treated with EV. It should be noted that in this work we register the complex effect of exosomes on gene expression in uninfected cells. In this case, not only RNA, but also proteins can be involved in the regulation of the immune response of cells, which requires further study.

## Figures and Tables

**Table 1:** Primer and TaqMan probe sequences.

Gene	Forward Primer	Reverse Primer	Probe
*IFIT1*	AAACTTCGGAGAAAGGCATTAGAT	TGAAATGAAATGTGAAAGTGGCTG	(HEX)-CCTGAGACTGGCTGCTGACTTTGAGAAC-(BHQ1)
*MxA*	GAGACAATCGTGAAACAGCAAATCA	TATCGAAACTCTGTGAAAGCAAGC	(HEX)-CACTGGAAGAGCCGGCTGGGATATG-(BHQ1)
*RIG1*	GAGCACTTGTGGACGCTTTA	ATACACTTCTGTGCCGGGAG	(ROX)-CCTGGCATATTGACTGGACGTGGC-(BHQ2)
*MDA5*	AAACCCATGACACAGAATGAACA	TGTGAGCAACCAGGACGTAG	(Cy5.5)-CACAGTGGCAGAAGAAGGTCTGGA-(BHQ3)
*SOCS1*	CCTGGTTGTTGTAGCAGCTTA	CCTGGTTTGTGCAAAGATACTG	(ROX)-CCTGGTTGTTGTAGCAGCTTA-(BHQ2)
*PKR*	GAAAGCGAACAAGGAGTAAGGGA	CCATCCCGTAGGTCTGTGAAA	Cy5-AGCCCCAAAGCGTAGAGGTCCACTTCC-BHQ3
*NFκB*	GCTCAGTGAGCCCATGGAAT	TGATGCTCTTGAAGGTCTCATATGTC	(FAM)-TCACCGGATTGAGGAGAAAC-(BHQ1)
*COX2*	CTGATGATTGCCCGACTCCC	GGCGCAGTTTACGCTGTCTA	(ROX)-GGGCTGGGCCATGGGGTGGA-(BHQ2)
*AnxA1*	ACCACCAGAAGCTATCCACAA	CGAGTTCCAACACCTTTCATGG	(Cy5.5)-AAGTGCGCCACAAGCAAACCAGC-(BHQ3)
*GAPDH*	CAGTCAGCCGCATCTTCTTTTGCGTCG	CAGAGTTAAAAGCAGCCCTGGTGACCAG	(FAM)-TGGGGAAGGTGAAGGTCGGAGTCAACGGATTTGGTC-(BHQ1)
*IL6*	CCACTCACCTCTTCAGAACG	CATCTTTGGAAGGTTCAGGTTG	(HEX)-AAATTCGGTACATCCTCGACGGCATC-(BHQ1)
*IL18*	AAACTATTTGTCGCAGGAATAAAGAT	GCTTGCCAAAGTAATCTGATTCC	(ROX)-TGCAATTGTCTTCTACTGGTTCAGCAGC-(BHQ2)
*NS*	TACCTAACTGACATGACTCTTGAG	TCGCCTGGTCCATTCTGATAC	-
*M2*	GACCRATCCTGTCACCTCTGAC	AGGGCATTYTGGACAAAKCGTCTA	(FAM)-TGCAGTCCTCGCTCACTGGGCACG-(BHQ1)
*PA*	TTCAGGCACTTAGGGACAA	AGGAAGGAGTTGAACCAAGA	(HEX)-TGCCTGATTAATGATCCCTGGGTTTTGC-(BHQ1)

**Table 2:** RT-PCR cycle thresholds.

Sample	RT Primers	GAPDH	IAV Genes		
			*M2*	*NS*	*PA*
**EV**	*dT16*	37.7	38.28	38.43	N/A
	*Random9*	38.55	32.33	30.83	36.29
**E**	*dT16*	38.76	N/A	N/A	N/A
	*Random9*	37.4	N/A	N/A	N/A

## Data Availability

The data that support the findings of this study are available from the corresponding author, Y.Z., upon reasonable request.
